# A narrative synthesis of the impact of primary health care delivery models for refugees in resettlement countries on access, quality and coordination

**DOI:** 10.1186/1475-9276-12-88

**Published:** 2013-11-07

**Authors:** Chandni Joshi, Grant Russell, I-Hao Cheng, Margaret Kay, Kevin Pottie, Margaret Alston, Mitchell Smith, Bibiana Chan, Shiva Vasi, Winston Lo, Sayed Shukrullah Wahidi, Mark F Harris

**Affiliations:** 1 Centre for Primary Health Care and Equity, University of New South Wales, Sydney, Australia; 2 Southern Academic Primary Care Research Unit, School of Primary Health Care, Faculty of Medicine, Nursing and Health Sciences, Monash University, Melbourne, Australia; 3 Discipline of General Practice, The University of Queensland, Brisbane, Australia; 4 Department of Family Medicine, and Department of Epidemiology & Community Medicine, The University of Ottawa; Canadian Collaboration for Immigrant and Refugee Health, Ottawa, Canada; 5 Department of Social Work, Monash University, Melbourne, Australia; 6 New South Wales Refugee Health Service, South Western Sydney Local Health District, Sydney, Australia; 7 School of Public Health & Community Medicine, The University of New South Wales, Sydney, Australia

**Keywords:** Access, Coordination, Health care models, Primary health care, Quality of care, Refugee, Migrant, Immigrant, Health services evaluation

## Abstract

**Introduction:**

Refugees have many complex health care needs which should be addressed by the primary health care services, both on their arrival in resettlement countries and in their transition to long-term care. The aim of this narrative synthesis is to identify the components of primary health care service delivery models for such populations which have been effective in improving access, quality and coordination of care.

**Methods:**

A systematic review of the literature, including published systematic reviews, was undertaken. Studies between 1990 and 2011 were identified by searching Medline, CINAHL, EMBASE, Cochrane Library, Scopus, Australian Public Affairs Information Service – Health, Health and Society Database, Multicultural Australian and Immigration Studies and Google Scholar. A limited snowballing search of the reference lists of all included studies was also undertaken. A stakeholder advisory committee and international advisers provided papers from grey literature. Only English language studies of evaluated primary health care models of care for refugees in developed countries of resettlement were included.

**Results:**

Twenty-five studies met the inclusion criteria for this review of which 15 were Australian and 10 overseas models. These could be categorised into six themes: service context, clinical model, workforce capacity, cost to clients, health and non-health services. Access was improved by multidisciplinary staff, use of interpreters and bilingual staff, no-cost or low-cost services, outreach services, free transport to and from appointments, longer clinic opening hours, patient advocacy, and use of gender-concordant providers. These services were affordable, appropriate and acceptable to the target groups. Coordination between the different health care services and services responding to the social needs of clients was improved through case management by specialist workers. Quality of care was improved by training in cultural sensitivity and appropriate use of interpreters.

**Conclusion:**

The elements of models most frequently associated with improved access, coordination and quality of care were case management, use of specialist refugee health workers, interpreters and bilingual staff. These findings have implications for workforce planning and training.

## Introduction

Ensuring effective primary health care (PHC) for refugees is an increasing concern globally. In 2011, developed countries worldwide received 441,448 applications for refugee status, a 20% increase from the previous year [1]. A refugee is a person outside the country of his or her nationality who, “owing to a well-founded fear of being persecuted for reasons of race, religion, nationality, membership of particular social group or political opinion, is outside the country of his nationality and is unable or owing to such fear unwilling to avail himself of the protection of that country” [2]. The refugee community in Australia is culturally and linguistically diverse, originating from more than 40 different countries [1]. These refugees are racially, culturally and linguistically diverse and often have suffered extreme mental and physical trauma [3], coming from countries in situations of long-term war or conflict [4].

Refugees often have complex or multiple health care needs as a consequence of inequities in the social determinants of health: experiences of persecution, torture and other forms of trauma, deprivation, unhealthy environmental conditions and disrupted access to health care [5]. PHC services need to be able to meet these challenges both on refugees’ arrival and in their transition to long-term care [3], providing culturally appropriate and timely care [6].

Refugees are more likely to have increased morbidity, poor health habits and a decreased life expectancy [4].They may have difficulty navigating the new education, housing, social support services and health systems in their country of resettlement [7]. Limited local language proficiency has an impact on health [8] and on the quality and accessibility of care [9]. It also influences access to the resources required for health, such as education, employment and social support [8]. When several service providers are involved, poor integration reduces their ability to deliver care effectively and efficiently [10]. There is inadequate community support for refugees in moving between services and sectors [11,12].

Enabling refugees to access timely, high-quality health care is crucial to their successful settlement and integration, as optimal health and well-being provide a stronger basis for them to adapt and thrive in their new country [11,13]. Good physical and mental health are vital for refugees to deal effectively with the challenges of settling in a new country and to participate fully in economic, social and cultural life [12]. Although other population groups face access barriers, the diverse and complex health and well-being needs of people from refugee backgrounds require specific attention [14-16].

Diverse initiatives have been instituted by governments and by non-government organisations to address the needs of refugee populations in countries of resettlement. This review focuses on PHC service delivery models for refugee populations and the impacts of components of these models on access, coordination and quality of care in countries of resettlement, with the aim of informing refugee health policy and its implementation.

## Method

A narrative systematic review was conducted. Medline, CINAHL, EMBASE, Cochrane Library, Scopus, Australian Public Affairs Information Service – Health, Health and Society Database, Multicultural Australian and Immigration Studies and Google Scholar were searched from February to March, 2012 for articles that included “refugee”, “primary health care” and “model of care” (or associated terms) in the title, keywords or abstract (Appendix 1). In Medline, the following search terms were used in the titles, abstract and keywords: (“Primary Health Care” OR “General Practice” OR “Comprehensive Health Care” OR “Physician, Family” OR “Family Practice” OR “Family Medicine” OR “Community Health Services”) AND (“Refugee” OR “Refugees” OR “Transients and Migrants” OR “Emigration and Immigration” OR “Asylum Seeker”) AND (“Model of Care” OR “Long-Term Care” OR “Models, Organisational” OR “Continuity of Patient Care” OR “Delivery of Health Care” OR “Patient Care Team”). The “explode” option was used to increase the depth of the search.

This was followed by a limited snowballing exercise, searching the reference lists of all included studies. Further studies and studies from the grey literature were identified with the assistance of the stakeholder advisory committee and international advisers and networks in Australia and overseas. These studies included reports on websites of key government (USA, Australia and Canada), international bodies (World Health Organization, World Bank, International Organization for Migration, United Nations High Commissioner for Refugees) and non-government organisations (The Victorian Foundation for Survivors of Torture, Boston Center for Refugee Health & Human Rights, Canadian Collaboration for Immigrant and Refugee Health, Ontario Multicultural Health Applied Research Network, and the Welcoming Communities Initiative). Systematic reviews fulfilling the selection criteria were also included in the review. The PRISMA checklist was followed for reporting [17].

**Figure 1:**
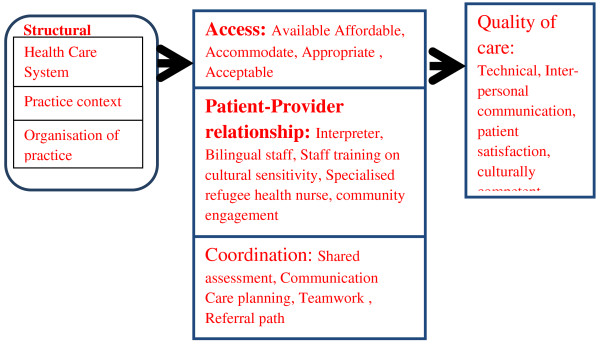
**Framework describing impact of primary health care service delivery models (adapated from Hogg et al.) [**[29]**].**

Studies were included in the review if they were about PHC in the major developed countries accepting refugees for long-term resettlement (Australia, USA, Canada, Sweden, Norway, New Zealand, Finland, Denmark, Netherlands and the UK) [18] and were published in English between 1990 and 2011. Inclusion criteria were that studies evaluated models of care that included specific aspects of care for refugee populations and the organisation and/or delivery of PHC. We did not predefine the way in which these models were evaluated and we accepted measures of the outcomes based on service provider or client assessment using either qualitative or quantitative methods. Table 1 provides the key definitions used in the review. Figure 1 shows a framework for thinking about how these relate to each.

**Table 1:** Key definitions used in the review

**Terms**	**Definitions**
Refugee	A refugee is a person forced to flee his or her home due to a well-founded fear of being persecuted for reasons of race, religion, nationality, membership of a particular social group or political opinion, and who is unable or unwilling to return to his or her country of origin [2]. This includes humanitarian refugees with permanent residency visas, refugee asylum seekers (in community and detention), refugees with temporary protection visas. This review is primarily focused on refugees whose time since arrival in their country of resettlement is less than10 years.
Primary health care	Primary care is the level of the health service system “that provides entry into the system for all new needs and problems. Primary care provides person-centred care over the continuum of time, assistance for all common conditions, and co-ordinates and integrates care provided by others” [19]. We take PHC to include care provided in the community settings through general practice, private and publicly funded community, allied health and nursing services and non-government organisations. Activities carried out in PHC include:
	• Assessment of health on arrival, including identification of infectious disease, mental health
	• Ongoing management of acute or chronic illnesses, mental illnesses, psychosocial illnesses
	• Provision of preventive care
	• Referral to or links with more specialised medical services
	• Referral, links to or provision of social care, housing, employment, education, or legal advice.
Model of care	A model of care describes the way in which a complex range of health services are organised and delivered [20]. This may be defined by principles (such as equity, accessibility, comprehensiveness, coordination), care delivery systems (e.g. multidisciplinary, on-line, the nature of consumers and the pathway of care which they must negotiate (e.g. entry, referral, etc.) and the range of services provided (e.g. medical specialist, generalist). These are underpinned by organisational and infrastructural elements which include:
	• health service funding/cost to clients/system: government, non-government organisation, private
	• provider workforce: e.g. general practitioners, nurses, social workers, allied health
	• organisation: team, network, integrated service
Access to the service	Access is the opportunity or ease with which consumers or communities are able to use appropriate services in proportion to their need [21]. As such it is influenced by both provider and consumer characteristics. Andersen described a model in which health care utilisation was determined by population and health system characteristics and influenced by patient satisfaction and outcomes [22]. The characteristics of PHC which determine their accessibility have been described by Pechansky (1981) [23] and more recently by Gulliford et al. [24] as:
	• Availability of a sufficient volume of services (including professionals, facilities and programmes) to match the needs of the population and the location of services close to those needing them
	• Affordability (cost versus consumers’ ability to pay, impact of health care costs on socioeconomic circumstances of patients)
	• Accommodation – the delivery of services in such a manner that those in need of them can use them without difficulty (e.g. appropriate hours of opening, accessible buildings)
	• Appropriateness to socioeconomic, educational, cultural and linguistic needs of patients
	• Acceptability in terms of consumer attitudes and demands
Coordination of care	This involves coordination of care between multiple providers and services with the aim of achieving improved quality of care and common goals for patients [25]. It may involve
	• Care planning
	• Informal communication between workers or services
	• Team meeting, case conferences, interagency meetings
	• Shared assessments and records
	• Coordination with non-health services including language services (interpreters, translated health information), formal settlement services, torture and trauma services
	• Referral pathways and inter-service agreements
Quality of care	We define quality of care as the consistency of clinical care with recommendations in evidence-based guidelines as well as the quality of interpersonal care [26]. This includes patients’ satisfaction with aspects of care [27]. The Institute of Medicine has defined health care quality as the extent to which health services provided to individuals and patient populations improve desired health outcomes. The care should be based on the strongest clinical evidence and provided in a technically and culturally competent manner with good communication and shared decision making [28]. It includes technical quality of primary and secondary prevention, and the management of chronic and acute conditions [29].
Case management	Case management has been variously defined. In this study we defined it as a collaborative process of assessment, planning, facilitation, care coordination, evaluation, and advocacy for options and services to meet an individual’s and family’s comprehensive health needs through communication and available resources to promote quality cost effective outcomes [30]

The papers were screened initially by CJ and a 20% random sample of excluded studies was reviewed and checked by MFH. Consensus was gained for uncertain articles through discussion between MFH and CJ. Included studies were screened based on the title and abstract and the full paper verified through assessment of its contents based on the inclusion criteria. Data extraction, including a quality assessment using a published checklist [31] was carried out by CJ and a 20% sample was verified by MFH. The extracted data were entered in an MS-Excel spread-sheet and included variables such as country of resettlement and country of origin, years in the destination country, location (urban or rural), characteristics of participants including type of refugee, country of origin, years in country, age, gender and major health problems or concerns. Also recorded was information about the study design, methodology and quality, types of service, model of care and the impact of services including access, coordination, quality of care, costs involved and the health outcomes of interventions. Because the outcomes were heterogeneous a meta-narrative synthesis was undertaken [32].

## Results

**Figure 2:**
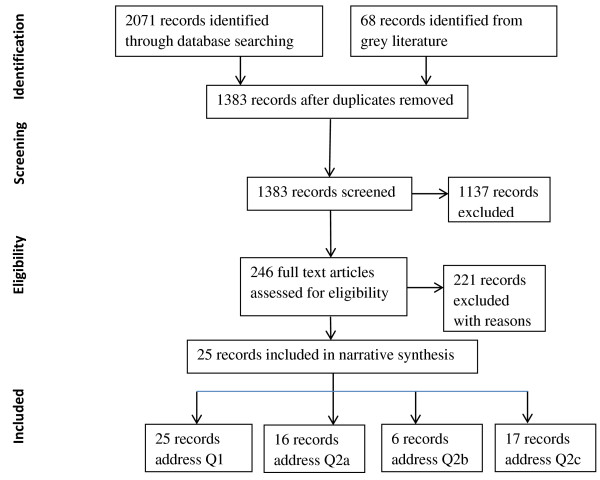
PRISMA flow diagram on selection of papers for the review.

The database searches and grey literature produced 2,139 papers which were assessed for inclusion in the review. We found relatively few evaluated models of refugee health care in countries of resettlement. Most evaluations focused on patient satisfaction rather than other outcomes. After the screening and verification stages, data were extracted from 25 studies that described evaluated models of PHC for refugees. The major reasons for exclusion were that the paper did not report empirical research, was not from the predefined countries of resettlement, was not based on primary health care or lacked evaluation of the models. Figure 2 shows a PRISMA flowchart of the selection process. The models were then analysed according to their impact on access, coordination and quality of care (Table 2).

**Table 2:** Characteristics of the models and their impacts

**Study**	**Country of study**	**Components of the model**	**Impacts**
**Studies on access to health care only**			
Cheng et al. 2011	Australia	*Staff*: Refugee health nurse, volunteers, multidisciplinary, multilingual.	Increased utilisation of services.
		*Services*: Orientation on health care system, interpreting, mental health, dental health, eye health, audiology, outreach, health checks, referral pathways, partnership, case management, care plan, medical specialist referral, accommodation.	
Sypek et al. 2008	Australia	*Services*: Mental health, accommodation.	Barriers: cost, interpreter access, bulk billing doctors, unmet mental health needs, dental health and specialised auditory treatment.
Geltman and Cochran 2005	United States	*Staff*: Network of providers with enhanced knowledge on refugee health.	Timely health screening.
		*Services*: health screening and specialised medical service.	
Eytan et al. 2002	Switzerland	*Services*: Health screening and interpreting	Increased referral to medical and psychological care.
Ford 1995	United States	*Staff*: Bilingual staff,	Increased use of preventive and curative care.
		*Services*: Outreach, referral pathway, no cost/subsidized, interpreting, health screening and immunization.	
**Studies on coordination of care only**			
Mitchell 1997	Australia	*Staff*: Multidisciplinary.	Good coordination among service providers.
		*Services*: Accommodation, patient advocacy, interpreting, health education and orientation about the Australian Healthcare system, mental health support, case management, outreach, medical specialist referral.	
**Studies on quality of care only**			
Grigg-Saito et al. 2010	United States	*Staff*: Interpreting, cultural competency training to staff, outreach.	Improved physical and mental health status.
		*Services*: bilingual community health workers, multidisciplinary.	
Gould et al. 2010	Australia	*Staff*: Multidisciplinary, network of providers.	Timely medical care.
		*Service*: Health screening, referral to specialists, interpreting, no cost, dental health, transport, orientation on health care system.	
Birman et al. 2008	United States	*Staff*: Multidisciplinary and bilingual.	Improved mental health.
		*Services*: Mental health, case management, patient advocacy, referral, interpreting, transport, outreach.	
Goodkind 2005	United States	*Staff*: Students.	Improved mental health.
		*Services*: Mental health, interpreting, outreach.	
Fox et al. 2005	United States	*Staff:* Bilingual.	Improved mental health.
		*Services:* Mental health.	
Barrett et al. 2000	Australia	*Staff*: Bilingual.	Service culturally acceptable, reduced levels of anxiety.
		*Services*: Mental health, interpreting.	
Clabots and Dolphin 1992	United States	*Services*: Multilingual video tapes to provide health education and information on how to access the health care system	Culturally sensitive and appropriate for clients.
**Studies on access and coordination of care**			
Australian Resource Centre for Healthcare Innovations 2009	Australia	*Staff*: Network of providers with enhanced knowledge on refugee health, multidisciplinary, refugee health nurse.	Improved access to preventive care (health checks and immunisation), improved communication and coordination between providers.
		*Services*: Education and information, partnership, referral pathway, case management, health checks, medical specialist referral, immunization and preventive care.	
Kelly 2008	Australia	*Staff*: Refugee health nurse.	Improved access to primary health care and specialist services, increased number of patients from refugee backgrounds, good coordination among services.
		*Services*: Outreach, no or low cost, dental health, optometry, transport, patient advocacy.	
**Studies on access and quality of care**			
Department of Health and Human Services, 2010	Australia	*Staff*: multilingual staff, refugee health nurse.	Increased use of interpreters and culturally aware staff.
		*Services*: Education and information, interpreting, community advocacy, case management, mental health, health screening, referral pathways, specialist medical treatment.	Difficulty accessing refugee health nurse, bicultural workers, culturally appropriate interpreters and mental health services.
Companion House 2009	Australia	*Services*: Mental health.	Improvement in mental health, difficulty accessing medicines due to cost.
Sheikh and MacIntyre 2009	Australia	*Services*: Media awareness of health service, health education.	Increased clinic attendance and enhanced knowledge on preventive care.
Smith 2009	Australia	*Staff*: Refugee health nurse, multilingual staff, both male and female GPs.	Client satisfaction with multilingual staff. Ineffective referral to non-health services, lack of mental health service, non-representative interpreters.
		*Services*: Patient advocacy, interpreting, case management, outreach, health education, transport, education and information, partnership, dental health and allied health.	
O’Donnell et al. 2007	United Kingdom	*Staff*: Asylum support nurse for coordination with health service and conducting health checks.	Increased GP registration, trust built between patients and health services.
Pottie and Hosland 2007	Canada	*Staff*: Medical students.	Patient satisfaction, increase in trust between patients and health care providers, increased knowledge of health system and easier access. Interpreter service was not reliable.
		*Services*: Orientation on health care system, outreach, health education, students trained in cultural sensitivity, health and social support.	
Samaan 2004	Australia	*Staff*: Volunteers.	Client satisfaction with onsite interpreters and patient advocacy.
		*Services*: Outreach, interpreting services, transport, patient advocacy, longer consultation sessions with GP, partnership, health check, immunisation, mental health, dental health, eye health, allied health, case management, no cost, referral pathways.	
			Barriers: cost, lack of local transport
			Interpreter access, non-representative interpreting, lack of bulk billing doctors, difficulty in physical access for people with disabilities and remote location.
**Studies on access, coordination and quality of care**			
Department of Health 2011	Australia	*Staff*: Refugee health nurse.	Enhanced access to services, culturally appropriate service, good coordination among services and continuum of care.
		*Services*: Mental health, dental health, eye health, health assessment, referral to specialist services, English classes, interpreters, accommodation.	
Robson 2011	Australia	*Staff*: Refugee health nurse.	Client satisfaction, staffs of other organisation confident on coordinating care with the centre, increased access to preventive care.
		*Services*: Outreach, patient advocacy, partnership, referral pathway, medical specialist referral, health screening, immunisation, case management, health education, optometry, audiology, mental health, dental health, allied health.	
Western Region Health Centre 2001	Australia	*Staff*: Refugee health nurse.	Clients satisfied about information on accessing different services including transport.
		*Services*: Partnerships, orientation on the health care system, information on rights, entitlements and services available and how to access them, longer consultation, training in cultural sensitivity to staff, interpreting, referral pathway, allied health.	Problems with cultural competency in spite of receiving training, time management for staffs due to longer consultations and dissatisfaction with long waiting time, inadequate follow up, unnecessary referrals in absence of interpreters, interpreting service non-representative.
			Coordination with some service providers was good while there was a lack of coordination with many of them.

### Models of care

There were 10 overseas and 15 Australian models of PHC for refugees. The characteristics of the models were described under six categories: service context, clinical model, workforce, cost to clients, health services and non-health services (Table 3). Case management and care planning were common components of the clinical models evaluated. In Australia, the majority of models used case management to coordinate a range of health (especially mental health, specialised services and access to general practitioners) as well as non-health services [20,33-40]. Clinical services were often provided by a multidisciplinary team [20,33,37,40-42]. Several studies described the role of specialist refugee health nurses in assessment and care coordination [20,33,34,36,38,39,43,44]. Those roles routinely included assessment of health and social needs, immunisation, and case management of referral to other services and ongoing liaison and transfer to GPs. Training (including training in cross-cultural communication) underpinned the capacity of many health staff to provide appropriate care [30,33,34]. The provision and use of interpreters and bilingual staff were key components of many models [33,35-37,39,42,45-48]. In Australia, interpreters and/or bilingual staff were used in most services. Outreach into the homes of refugees or the community with a comprehensive range of service was another commonly described approach [33-36,40,42,44,46,49,50]. These services were delivered by a range of health professionals including health visitors, students and ethnic health workers. In Australia, outreach was a common model provided in conjunction with case management by specialist refugee health nurses.

**Table 3:** Characteristics of models of PHC for refugees and their corresponding evaluated components

**Main characteristics**	**Evaluated components of the models**		**List of studies**
Service context	Organisational	Specialist service	Ford et al. 1995
		Part of a hospital	Sypek et al. 2008; Samaan 2004
	Location	Urban	Cheng et al. 2011; Department of Health 2011; Grigg-Saito et al. 2010; Australian Resource Centre for Healthcare Innovations 2009; Sheikh & MacIntyre 2009; Smith 2009; Birman et al. 2008; Fox et al. 2005; Eytan et al. 2002; Western Region Health Centre 2001; Mitchell 1997; Clabots and Dolphin 1992
		Rural	Gould et al. 2010; Sypek et al. 2008
		State	Department of Health and Human Services 2010; Samaan 2004
	Partnerships		Cheng et al. 2011; Robson 2011; Australian Resource Centre for Healthcare Innovations 2009; Smith 2009; Samaan 2004; Western Region Health Centre 2001
	Media		Sheikh & MacIntyre 2009
Clinical model	Case management		Robson 2011; Department of Health and Human Services 2010; Australian Resource Centre for Healthcare Innovations 2009; Smith 2009; Birman et al. 2008 Samaan 2004; Cheng et al. 2011; Western Region Health Centre 2001; Mitchell 1997
	Care planning		Cheng et al. 2011
	Outreach		Cheng et al. 2011; Robson 2011; Grigg-Saito et al. 2010; Smith 2009; Birman et al. 2008; Kelly 2008; Pottie & Hosland 2007; Goodkind 2005; Samaan 2004; Mitchell 1997; Ford et al. 1995
	Health checks		Cheng et al. 2011; Robson 2011; Australian Resource Centre for Healthcare Innovations 2009; O’Donnell et al. 2007
	Referral pathways		Cheng et al. 2011; Robson 2011; Australian Resource Centre for Healthcare Innovations 2009; Samaan 2004; Western Region Health Centre 2001; Ford et al. 1995
Workforce	Specialised workers (refugee nurses)		Cheng et al. 2011; Robson 2011; Department of Health and Human Services 2010; Australian Resource Centre for Healthcare Innovations 2009; Smith 2009; Kelly 2008; O’Donnell et al. 2007; Western Region Health Centre 2001
	Training (cross-cultural)		Grigg-Saito et al. 2010; Pottie & Hosland 2007; Western Region Health Centre 2001
	Bilingual workers, interpreters		Cheng et al. 2011; Department of Health and Human Services 2010; Grigg-Saito et al. 2010; Smith 2009; Birman et al. 2008; Fox et al. 2005; Goodkind 2005; Samaan 2004; Eytan et al. 2002; Barrett et al. 2000; Mitchell 1997; Ford et al. 1995; Clabots and Dolphin 1992
	Students and volunteers		Cheng et al. 2011; Pottie & Hosland 2007; Goodking 2005; Samaan 2004
Cost to clients	No-cost or subsidised		Gould et al. 2010; Kelly 2008; Samaan 2004; Ford et al. 1995
Health Services	Screening/prevention		Robson 2011; Department of Health and Human Services 2010; Gould et al. 2010; Geltman and Cochran 2005; Samaan 2004; Ford et al. 1995
	Mental health		Cheng et al. 2011; Robson 2011; Department of Health and Human Services 2010; Companion House 2009; Birman et al. 2008; Sypek et al. 2008; Fox et al. 2005; Goodkind 2005; Samaan 2004; Barrett et al. 2000
	Dental health		Cheng et al. 2011; Department of Health 2011; Robson 2011; Gould et al. 2010; Smith 2009; Kelly 2008; Samaan 2004
	Physical: general practitioner, eye, maternal and child health, infectious disease/immunisation		Cheng et al. 2011; Department of Health 2011; Robson 2011; Australian Resource Centre for Healthcare Innovations 2009; Sheikh & MacIntyre 2009; Kelly 2008; Samaan 2004; Western Region Health Centre 2001; Ford et al. 1995
	Allied health		Cheng et al. 2011; Robson 2011; Smith 2009; Samaan 2004; Western Region Health Centre 2001
	Medical specialist referral		Cheng et al. 2011; Department of Health 2011; Robson 2011; Gould et al. 2010; Australian Resource Centre for Healthcare Innovations 2009; Mitchell 1997
	Health education		Robson 2011; Sheikh & MacIntyre 2009; Smith 2009; Pottie and Hosland 2007; Mitchell 1997; Clabots and Dolphin 1992
Non-health services	Transport		Gould et al. 2010; Smith 2009; Birman et al. 2008; Kelly 2008; Samaan 2004;
	Housing		Cheng et al. 2011; Department of Health 2011; Sypek et al. 2008; Mitchell 1997
	Education and information		Cheng et al. 2011; Department of Health and Human Services 2010; Gould et al. 2010; Australian Resource Centre for Healthcare Innovations 2009; Smith 2009; Pottie & Hosland 2007; Western Region Health Centre 2001; Mitchell 1997; Clabots and Dolphin 1992
	Patient advocacy		Robson 2011; Department of Health and Human Services 2010; Smith 2009; Birman et al. 2008; Kelly 2008; Samaan 2004; Mitchell 1997

Mental health care (including counselling, cognitive behavioural therapy and psychiatry) was the most frequent health service described [33-35,37,39,40,43,45,48,49],[51,52]. Screening and assessment were described in several studies [34,39,41,46,47,53]. Child health was the focus in several models [37,45,54]. A number of studies described models involving the provision of both health and non-health services (e.g. legal, housing, education, transport) [20,33-41,43,44,50,51]. In Australia, many of the models involved screening or assessment and health as well as non-health services (often using a case management approach and in conjunction with settlement services).

Free transport to health centres was provided by many Australian models [35-38,41,44]. Low- or no-cost care for patients was provided in a number of overseas and Australian models through use of government insurance-only payments or volunteers [35,41,44,46,51]. Negotiation of *pro bono* medical services and volunteers was a feature of many Australian and US models [33,35,41,46].

### Impact on access

Sixteen studies evaluated the impact of the models of care on access to health care [6,20,33-36,38,39,43,44,46],[47,51-54]. The models broadly addressed several components: affordability, appropriateness and acceptability of primary care, and a variety of health and non-health services. Strategies used to enhance access included multidisciplinary staff (medical, nursing, allied health, non-health workers), use of interpreters and bilingual staff, no-cost or low-cost services to consumers, outreach services (many in refugees’ homes), free transport for appointments, longer consultation hours, patient advocacy and use of gender-sensitive providers. All these strategies had a positive impact on client satisfaction and increased utilisation of services. Other strategies to contain costs were the use of volunteers and minimising fees so that they were covered by government health insurance. Staff advocating on behalf of patients increased their access to housing, social security payments and medical services. Gender-sensitive providers helped overcome cultural barriers that prevented women from accessing health services. Teaching patients how to navigate the health care system led to better understanding and increased utilisation of health services. Bilingual and culturally appropriate information provision in written and video form enhanced health literacy and access to care. However, lack of interpreters in needed languages, unmet health needs, and shortage of doctors willing to accept fees limited to government insurance levels remained major barriers.

### Impact on coordination

Six studies [20,34,38,40,43,44] evaluated the impact of models of refugee care on coordination of health care services. A number of studies described models involving the provision of both health and non-health services (including legal assistance, housing, education and transport). Coordination between the different health care services and services responding to the social needs of clients was most frequently addressed through case management conducted by a refugee health nurse or other health professional and often involved visiting refugee clients in their home in the community. Team coordination, especially across agencies, was also used. These interventions were associated with improved communication and coordination between service providers, as well as improved access to preventive health services. Four of the six case management models reported improved outcomes. Individual case managers provided easier transition between the primary health care and hospitals [20], good coordination among stakeholders [43] and improved team work where workers alerted each other of patient issues [40]. Please refer to Table 2 for details. Two Australian studies without improved outcomes reported that coordination was compromised by limited access to services by some groups of patients and by the capacity of staff to meet the needs of patients [35,38]. The study using a multidisciplinary team approach to coordination reported that services were able to meet clients’ needs [36].

### Impact on quality of care

Seventeen studies evaluated the impact of models on quality of care [6,34-39,41-43,45,48-50,52,54,55]. The most frequently cited interventions were training providers in culturally sensitive care, appropriate use of interpreters, and bilingual staff. These resulted in improved client satisfaction, increased reporting of physical and psychological symptoms by the patients, improved referrals, improved physical and mental health, and increased access to health services. There were marked reduction in risk related to medical interpreters’ service [42], and increased access to culturally appropriate services [43]. Nonetheless, many patients continued to experience barriers, including persisting cultural and language barriers [36,38,39]. There were also difficulties reported in putting cultural sensitivity training knowledge into practice [38].

## Discussion

The aim of this review was to identify evaluated models of PHC for refugees in countries of resettlement and to evaluate their impact on access, coordination of care and quality of care. Relatively few studies met the inclusion criteria. There were many similarities among the evaluated models, with some variation according to the context and resources available. Integration between the different health care services and services responding to the social needs of clients was most frequently addressed by a case management approach conducted by a refugee health nurse or other health professional and often involving home visiting refugee clients in their home in the community. Interpreters, bilingual staff, and training of staff in cross-cultural management were also used to facilitate access to and quality of health and social care.

Refugees need to be able to access the same primary care services as the local population. Thus all primary care services need to be prepared to deliver health care to refugees in their local area and some services should develop models of care specifically addressing the needs of refugees because of the demographics of their local communities. Clients of refugee-specific services need to be able to transition into ongoing mainstream PHC [56]. Yet their transition is influenced by factors including lack of knowledge about available services and/or how they work, language barriers, and lack of appropriate services. Refugees may also be reluctant to use existing services because of fear, distrust, negative experiences and lack of confidence, sociocultural barriers and political, economic and administrative constraints on access to the health services [57,58]. The models in our review aligned broadly with the elements of accessibility proposed by Penchansky and Thomas [59]:

• Increasing awareness and health literacy in using health services with interventions involving media and health education.

• Outreach to facilitate registration or clinic attendance.

• Improving acceptability and appropriateness through the use of interpreters and bilingual workers.

• Coordinating service networks (often facilitated by refugee health nurses) to improve access to range of services and to transport.

• Reducing cost to clients by use of *pro bono* providers or not using co-payments.

Coordination of care has been discussed in the literature describing models of care for refugees. For example, there has been debate about integration between government and non-government services, the need for refugee-specific health services and mainstream services (which may include medical specialists) and the balance of emphasis on initial assessment compared with providing ongoing long-term care [60,61]. In this review we found that coordination of care was largely focused on integrating care across the number of health and non-health services that might be involved. The two main coordination models were case management and team coordination, and these were associated with improved communication and coordination between service providers.

Good patient–provider communication is of paramount importance to quality of care. Patient dissatisfaction arises more frequently from poor communication than from medical errors [62], and language barriers degrade the quality of care, resulting in poorer health outcomes [63,64]. Culture frames the experience and expression of emotional distress and social problems [65]. Language barriers and the cultural complexity of assessing symptoms have been shown to prevent adequate diagnosis and treatment [65]. Several studies in our sample evaluated impacts on quality of care. These service models included use of interpreters, bilingual staff, cross-cultural training of staff and specialised refugee health nurses, and engagement with the community. They were associated with improvements in staff confidence, detection of problems at assessment, clients’ assessment of the quality of communication and interpersonal care. These measures are broadly consistent with international policy [57].

The review had some important limitations. Few studies in the international databases described evaluated models. Few studies described coordination tools or protocols and regional coordination. Many of the studies were identified from website search and from our key informants in Australia, the UK, Canada and New Zealand. Because the search of the grey literature was less systematic and reliant on key informants, it is possible that other international grey literature was missed. There were high levels of heterogeneity in the impacts and outcomes evaluated. This made meta-analysis impossible. Thus a qualitative approach was used to analyse and compare the studies. There was little information on the cost of services or models. This meant that we were unable to undertake any comparative cost analysis of models. There is a need for more rigorous evaluations, especially focused on the impact of innovative models on access and quality of care especially for women.

We found no evaluated model of PHC that specifically focused on women or men; hence it was not possible to conduct a gender analysis. Similarly, previous studies of migrant utilisation of health services have not included a focus on women [66].

Despite the limitations of the available evidence, there are some implications for policy and practice. Many of the models emerged in response to the needs of local communities. Therefore the effective components identified in this review need to be flexibly applied in the local context. For example, case management is commonly used as a component of models and appears to be broadly successful in improving access and coordination. This makes sense where there are relatively few refugees requiring case management and where the focus is on coordination between services and integration of the refugee into long-term care. It is, however, potentially expensive and the case coordinator needs to have some specialised training. Workforce planning and development in this field is thus important, especially for nurses. At present, the education and training available for the workforce in many countries remains limited [57]. Although specialised services and providers may be useful, especially for on-arrival assessment, other forms of service delivery based on and integrated with mainstream PHC services are needed.

The use of interpreters and bilingual workers is well-documented as essential in facilitating access to care and delivering quality care. Interpreter services can be on-site or through a telephone service, especially for more routine care [67]. Use of informal interpreters such as family members can undermine the quality of care [63]. Thus it is important to make interpreter services more available to assist in PHC for refugees, both in person and on the phone.

## Conclusion

Refugees experience unique health problems which can be met by PHC services. Relatively few studies have evaluated models of PHC delivery to the refugee community. Much of the literature currently focuses on describing the health problems or access barriers experienced by refugees. This study goes further in identifying the specific strategies required to provide accessible and well-coordinated care for refugees, such as case management, use of specialised staff, interpreters and outreach. These have financial and workforce implications and comparable evaluation data needs to be collected to allow comparison of the cost and effectiveness of different models.

## Appendix 1

### Search terms

*1. Medline*: 771 search result

PHC:

Primary health care mp. Or exp Primary Health Care/ or general practice mp. Or exp General Practice/ or exp Community Health Services/ or exp Comprehensive Health Care/ or exp Physicians, Family/ or exp Family Practice/ or family medicine mp.

Refugee:

Refugee mp. Or exp Refugees/ or exp “Transients and Migrants”/ or exp “Emigration and Immigration”/ or asylum seeker.mp.

Model of care:

Exp “Continuity of Patient Care”/ or exp “Delivery of Health Care”/ or exp Patient Care Team/ or model of care.mp. or exp Long-Term Care/ or exp Models, Organisational/

*2. CINAHL*: 561 search results (limiting search to 1990 - 2011)

PHC:

(MH “Community Health Centers”) OR (MH “Community Mental Health Services+”) OR (MH “Community Mental Health Nursing”) OR (MH “Health Information Networks”) OR (MH “Community Health Nursing+”) OR (MH “Community Health Services+”) OR (MH “Community Health Workers”) OR (MH “Community Networks”)

Refugee:

(MH “Transients and Migrants”) OR “transients and migrants” OR (MH “Refugees”) OR “refugee” OR “asylum seeker” OR (MH “Emigration and Immigration”) OR (MH “Immigrants, Illegal”) OR (MH “Immigrants+”)

Model of care:

(MH “Models, Psychological+”) OR (MH “Models, Educational”) OR (MH “Models, Structural+”) OR (MH “Multidisciplinary Care Team+”) OR (MH “Gender Specific Care”) OR (MH “Health Care Costs+”) OR (MH “Health Services Needs and Demand”) OR (MH “Health Care Delivery+”) OR (MH “Health Care Delivery, Integrated”) OR (MH “Nursing Care Plans+”) OR (MH “Nursing Care Delivery Systems+”) OR (MH “Outcomes (Health Care)+”) OR “model of care”

*3. Embase*: 578 search results

PHC:

exp primary health care/ or exp general practice/ or exp community health nursing/ or exp general practitioner/ or exp community care/ or exp family medicine/

Refugee:

asylum seeker*.mp. or “transients and migrants”.mp. or “emigration and immigration”.mp.

or exp refugee/ or refugee*.mp.

Model of care:

exp patient care/ or exp model/ or exp “organization and management”/ or model of care.mp. or exp health care delivery/ or exp medical care/

*4. Cochrane library*: 14 search results

primary care* or family medicine* or general practice* or community health*

model of care

*5. Scopus*: 62 results

“Primary care” or “primary health care”

Refugee* or “asylum seeker*”

*6. APAIS health*: 8 search results (limiting search to 1990-2011)

“primary care” or “primary health care” or “family medicine” or “general practice” or “community health” or “nursing servic*e” or “allied service*”

Refugee* or “asylum seeker*”

*7. Health and society database*: 31 search results

“primary care” or “primary health care” or “family medicine” or “general practice” or “community health” or “nursing service*” or “allied service*”

“asylum seeker’ or “refugee” or “refugees”

*8. MAIS (Multicultural Australian and Immigration Studies)*: 46 search results (limiting search to 1990-2011)

“primary care” or “primary health care” or “family medicine” or “general practice” or “community health” or “nursing service*” or “allied service*”

“asylum seeker” or “refugee” or “refugees”

## Abbreviations

PHC: Primary health care.

## Competing interests

The authors declare that they have no competing interests.

## Authors’ contributions

CJ identified the studies for inclusion in the systematic review, conducted the data extraction, assisted in writing up the review and drafted the manuscript. MH verified each process of the systematic review, prepared the review report and revised the manuscript. GR, I-HC, MK, KP, MS and SV suggested improvements for the review report and helped finalise the manuscript. MA and BC helped in revising the review report. All authors read and approved the final manuscript.
